# SEXATURN: sexual function of patients with Turner syndrome compared to that of women with premature ovarian insufficiency

**DOI:** 10.1093/sexmed/qfag074

**Published:** 2026-08-17

**Authors:** Eugénie de Paillette, Daphné Karila, Alexandra Rousseau, Emma Dubost, Tiphaine Meykiechel, Sophie Lamothe, Camille Vatier, Sylvie Jaillard, Mariana Nedelcu, Nathalie Bourcigaux, Bruno Donadille, Sophie Christin-Maitre

**Affiliations:** Centre of rare diseases CRESCENDO, Endocrine Unit, Hôpital Saint-Antoine, Assistance-Publique Hôpitaux de Paris, Endo-ERN Network, 75012 Paris, France; Centre of rare diseases CRESCENDO, Endocrine Unit, Hôpital Saint-Antoine, Assistance-Publique Hôpitaux de Paris, Endo-ERN Network, 75012 Paris, France; Plateforme de Recherche Clinique de l’Est Parisien, Unité de Recherche Clinique (URC)-Est, Hôpital Saint-Antoine, Assistance-Publique Hôpitaux de Paris, 75012 Paris, France; Centre of rare diseases CRESCENDO, Endocrine Unit, Hôpital Saint-Antoine, Assistance-Publique Hôpitaux de Paris, Endo-ERN Network, 75012 Paris, France; Centre of rare diseases CRESCENDO, Endocrine Unit, Hôpital Saint-Antoine, Assistance-Publique Hôpitaux de Paris, Endo-ERN Network, 75012 Paris, France; Centre of rare diseases CRESCENDO, Endocrine Unit, Hôpital Saint-Antoine, Assistance-Publique Hôpitaux de Paris, Endo-ERN Network, 75012 Paris, France; Centre of rare diseases CRESCENDO, Endocrine Unit, Hôpital Saint-Antoine, Assistance-Publique Hôpitaux de Paris, Endo-ERN Network, 75012 Paris, France; Sorbonne University, INSERM (National Institute for Medical Research in France) UMR 938, 75012 Paris, France; University of Rennes, CHU Rennes, Inserm, EHESP, Irset and Cytogenetic and cellular biology department (Institut de Recherche en Santé, Environnement et Travail) -UMR_S1085, Service de Cytogénétique et Biologie Cellulaire, 35000 Rennes, France; Centre of rare diseases CRESCENDO, Endocrine Unit, Hôpital Saint-Antoine, Assistance-Publique Hôpitaux de Paris, Endo-ERN Network, 75012 Paris, France; Centre of rare diseases CRESCENDO, Endocrine Unit, Hôpital Saint-Antoine, Assistance-Publique Hôpitaux de Paris, Endo-ERN Network, 75012 Paris, France; Centre of rare diseases CRESCENDO, Endocrine Unit, Hôpital Saint-Antoine, Assistance-Publique Hôpitaux de Paris, Endo-ERN Network, 75012 Paris, France; Centre of rare diseases CRESCENDO, Endocrine Unit, Hôpital Saint-Antoine, Assistance-Publique Hôpitaux de Paris, Endo-ERN Network, 75012 Paris, France; Sorbonne University, INSERM UMR 933, 75012 Paris, France

**Keywords:** Turner syndrome, primary ovarian insufficiency, sexuality, sexual dysfunction, physiological, female sexual function, female sexual function index, FSFI-6

## Abstract

**Introduction:**

Reduced sexual activity has been reported in women with Turner syndrome (TS), a chromosomal disorder frequently associated with premature ovarian insufficiency (POI), but data on sexual function and its qualitative aspects remain limited. Furthermore, few studies have compared sexual function of women with TS to that of women with POI from other etiologies (non-TS POI). Our aim was to assess sexual function in women with TS and compare it to that of women with non-TS POI, and to evaluate whether sexual dysfunction in TS is related to different comorbidities.

**Methods:**

SEXATURN is a monocentric, cross-sectional study (NCT 05223621) conducted at a French reference center for rare endocrine diseases. We included women aged 18–50 years with TS or non-TS POI. Sexual function was assessed using the abbreviated Female Sexual Function Index (FSFI-6). Clinical data were collected through questionnaires and electronic medical records. The primary outcome was the prevalence of impaired sexual function (FSFI-6 < 19). Secondary outcomes included comparison of FSFI-6 scores between TS and non-TS POI, FSFI-6 scores among sexually active patients and identification of factors associated with sexual dysfunction.

**Results:**

A total of 205 women were included (99 with TS, 106 with non-TS POI). Impaired sexual function was observed in 49.5% of women with TS and 43.4% of women with non-TS POI, without significant difference (*P* = .38). Among sexually active women, prevalence rates of impaired sexual function were similar (30.6% vs 31%) in both groups. Women with TS reported later sexual age of onset, fewer partners, and lower orgasm frequency. No association was found between sexual dysfunction and age, hormone replacement therapy nor TS-related comorbidities. Being single was strongly associated with impaired sexual function, particularly in women with TS (OR 8.82).

**Discussion:**

Strengths are a well-phenotyped cohort from a reference center, the use of a validated questionnaire and high response rates. Limitations include intrinsic limits of the FSFI-6, which does not assess distress or broader sexual experiences. SEXATURN study shows that women with TS exhibit reduced sexual activity but similar rates of impaired sexual function compared to women with non-TS POI. Being in a relationship is a protective factor. Our findings support the need of sexual and psychological care into multidisciplinary management of patients with TS.

**Trial registration:**

ClinicalTrial.gov (NCT 05223621) registration date January 24, 2022 and date of enrolment of the first subject December 02, 2022.

## Introduction

Turner syndrome (TS) is a rare chromosomal condition caused by the total or partial absence of an X chromosome, affecting about one in 2500 females.^1^ The main clinical features are short stature and premature ovarian insufficiency (POI), affecting 95% of patients.^2^ TS includes a range of congenital or acquired comorbidities, such as aortic or renal malformations, hearing loss, and autoimmune disorders. Patients with TS do not usually have intellectual disability, but may present mild learning difficulties in mathematics and weaknesses in visuospatial processing.^3^^,^^4^ Social interactions can be challenging due to a common psychosocial profile with a tendency to shyness and low self-esteem, a high prevalence of depression, anxiety disorders.^5^^,^^6^

POI is characterized by amenorrhea or oligomenorrhea for at least 4 months, before the age of 40, with Follicular Stimulating Hormone (FSH) higher than 25 IU/L and low estradiol.^7^ It affects 2%-4% of women. Different etiologies of POI have been reported besides TS, such as iatrogenic causes, pathogenic genetic variants, and autoimmune diseases.^8^^,^^9^ Nowadays, POI is idiopathic in 50%-60% of cases^10^ but whole exome sequencing analyses are progressively finding new genes.^11^^,^^12^

Several studies have found that sexuality is the second-most important concern after infertility in women with POI.^13–15^ So far, few studies have specifically explored the sexuality of patients with TS.^16^^,^^17^ TS patients have a later onset of sexual activity, less frequent intercourse, and fewer partners than the general population.^18–23^ However, most studies do not distinguish TS patients from patients with POI of other etiologies. Studies focusing on the quality of sexual life among TS patients are rare.^24^

The aim of the present study is to assess sexual function in a cohort of patients with TS, using the abbreviated Female Sexual Function Index score in 6 questions (FSFI-6), derived from the widely used FSFI score.^25^^,^^26^ Our main hypothesis was that sexual function is more impaired in patients with TS than in patients with non-TS POI. The remaining goals of our study were to answer two questions: (1) Are sexual function impairments secondary to TS per se? (2) What are the impacts of comorbidities, on sexual function, in women with TS?

## Subjects and methods

“SEXATURN” (SEXuAlity in patients with TURNer syndrome) is a monocentric, cross-sectional study including patients from a reference center for rare endocrine diseases of growth and development, located in a French teaching hospital, Hôpital Saint-Antoine, Assistance-Publique Hôpitaux de Paris, Sorbonne University, Paris. This center is affiliated to the “FIRENDO” national network for Rare Endocrine Diseases and to the European Union Reference Network of Rare Endocrine conditions (Endo-ERN network).

The study protocol was approved by a randomly assigned ethics committee from another institution on September 27, 2020 (IDRBC 2020-A01768-31). In compliance with French Law, the study complies with the reference methodology MR-003 published by the National Commission for Computing and Liberties (CNIL) and with the declaration of Helsinki. This study is registered on the website ClinicalTrial.gov (NCT05223621).

### Subjects

Women aged 18-50, with at least one visit, between 2018 and 2022, in our reference center, were eligible. Patients were included in the “TS group,” if their standard karyotype had at least 10% of cells displaying a total or partial loss of one X chromosome, according to the International recommandations.^1^ The “POI group” included patients with a karyotype ruling out TS. Exclusion criteria were being under guardianship or curatorship, aged under 18 or over 50. Absence of sexual activity, current or past, was not an exclusion criterion.

### Study protocol

The FSFI-6 is a validated short version of the FSFI, the most widely used questionnaire to assess sexual function.^25–27^ It includes six of the initial 19 questions, assessing six domains of sexuality during the 4 weeks preceding its completion: level of desire, level of arousal, frequency of vaginal lubrication, frequency of orgasm, overall satisfaction with sexual life, and frequency of pain. The total score ranges from 2 to 30. A score below 19 is in favor of an impairment of female sexual function.^26^

Eligible patients received an information note by post, before a phone call a week later for consent collection. Patients then received two questionnaires: the FSFI-6 and a questionnaire collecting epidemiological and medical data, including age, relationship status, number of children, highest degree obtained, employment status, past sexual intercourse, age at first intercourse, number of sexual partners, weight, height, age at menarche, pregnancy history and current use of hormonal replacement therapy (HRT). For the “TS group,” additional data included age at diagnosis of TS, history of growth hormone treatment and/or pubertal induction, history of cardiovascular diseases, autoimmunity, and hearing impairment.

In case of missing questionnaires, up to two follow-up phone calls were carried out.

The population analyzed consists of patients who returned both questionnaires with a fully completed FSFI-6.

Further data were obtained from the patients’ medical electronic record (Orbis software) such as age at POI and last prescription of HRT. In the “TS group,” data included karyotype, history of pubertal induction, history of cardiac or aortic pathology, last aortic root index and history of autoimmune disease. In the “POI group,” etiologies of POI were collected.

### Statistical analysis

Data management and statistical analysis were carried out by the Clinical Research Unit of the teaching hospital. *P*-values < .05 were considered statistically significant. No adjustment for multiplicity was made. Statistical analysis was conducted using SAS v9.4 (SAS Institute Inc) software.

Qualitative variables are described by their count and proportion; continuous variables by their mean and standard deviation (SD) or by their median and interquartile range (IQR), depending on the distribution of the variable.

The total FSFI-6 score, as well as each sub-score, is given for the “TS group” and the “POI group.” Scores are compared using a non-parametric test chosen based on the distribution. The proportion of women with impaired sexual function, defined as an FSFI-6 score below 19, was calculated using the exact Clopper-Pearson method, with a 95% confidence interval (CI95). The search for factors associated with a total FSFI-6 score below 19 was conducted using univariate and multivariate logistic regressions, with multiple imputation of missing values, using SAS procedures PROC MI and PROC MIANALYZE.

## Results

### Study population

**Figure 1:**
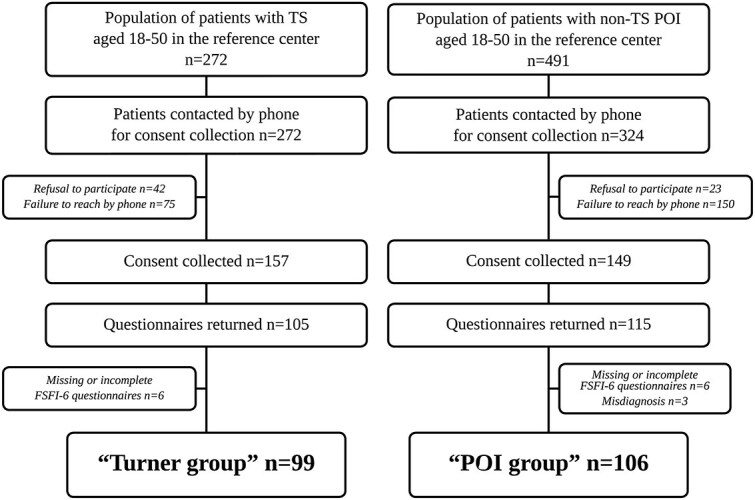
Flow chart of the patients from the reference center for rare endocrine diseases in SEXATURN study.

For the “TS group,” 272 patients were eligible and 491 for the “POI group” (Figure 1). A total of 306 patients agreed to participate and were included between December 2, 2022, and June 1, 2023. Among them, 220 returned the questionnaires: 105 (67%) from the “TS group” and 115 (77%) from the “POI group.” The last questionnaire was received on January 31, 2024. The final analysis included 205 completed questionnaires: 99 in the “TS group” and 106 in the “POI group.”

### Baseline characteristics

Characteristics of all women are presented in Table 1. As expected, women with TS were shorter than women with POI. At the time of the questionnaire, patients with TS were younger than those in the “POI group” (median age 35 years [IQR 28;41] vs 39 years [IQR 33;43], *P* = .018). Both groups had similar education levels and current employment situations. The percentage of patients on HRT was not statistically different between both groups. Patients with TS were less frequently in a relationship (*P* = .038).

**Table 1:** Baseline characteristics of the overall cohort.

	**Overall cohort n = 205**		**“TS group” n = 99**		**“POI group” n = 106**		***P*-value**
**Median age at completion of questionnaire (yr., IQR)**	205	37 [30;42]	99	35 [28;41]	106	39 [33;43]	.018^a^
**Relationship status (n, %)**	204		99		105		.038^b^
Single		70 (34.1)		41 (41.4)		29 (27.6)	
In a relationship		134 (65.4)		58 (58.6)		76 (72.4)	
**Highest diploma (n, %)**	203		98		105		.634^c^
No diploma		2 (1.0)		0 (0)		2 (1.9)	
General Education or lower		47 (23.2)		26 (26.5)		21 (20)	
Higher education diploma:		154 (75.9)		72 (73.5)		82 (78.1)	
2-years		28 (13.8)		11 (11.2)		17 (16.2)	
3-years		37 (18.2)		18 (18.4)		19 (18.1)	
4-years or more		89 (43.8)		43 (43.9)		46 (43.8)	
**Currently employed (n, %)**	204	168 (82.4)	99	81 (81.8)	105	87 (82.9)	.846^b^
**Median weight (kg, IQR)**	201	60 [52;70]	97	57 [49;66]	104	64.5 [58;74]	<.0001^a^
**Mean height (cm, SD)**	203	160 ± 9.2	98	154 ± 6.8	105	165.6 ± 7.5	<.0001^a^
**Median BMI (kg/m** ^**2**^ **, IQR)**	201	23.3 [21;27.3]	97	23.4 [21.2;27.5]	104	23.3 [20.4;27]	.717^a^
**Median age at menarche (yr., IQR)**	190	14 [12;15]	89	14 [13;16]	101	13 [12;14]	<.0001^a^
**Median age at POI diagnosis (yr., IQR)**	205		99	14.5 [13;16.3]	106	28 [20;34]	<.0001^a^
**Women with at least one child (n, %)**	204	70 (34.3)	98	18 (18.4)	106	52 (49.1)	<.0001^a^
**Women with a previous pregnancy (n, %)**	205	80 (39.0)	99	23 (23.2)	106	57 (53.8)	<.0001^b^
**Current HRT use (n, %)**	199	178 (89.4)	99	85 (85.9)	100	93 (93.0)	.101^b^
**Women with a history of sexual intercourse (n, %)**	205	177 (86.3)	99	78 (78.8)	106	99 (93.4)	.002^b^
**Median age at first intercourse (yr., IQR)**	174	19 [17;22]	77	20 [19;23]	97	19 [17;21]	.011^a^
**Number of partners (n, %)**	174		75		99		.025^b^
1-2		62 (35.6)		33 (44.0)		29 (29.3)	
3-5		57 (32.8)		27 (36.0)		30 (30.3)	
6-10		31 (17.8)		10 (13.3)		21 (21.2)	
>10		24 (13.8)		5 (6.7)		19 (19.2)	

Depending on the distribution of the variable, different tests were used:

a Wilcoxon-Mann–Whitney test.

b Chi-square test.

c Fisher’s test.

Abbreviations: TS = Turner syndrome, POI = Premature ovarian insufficiency, Yrs = years, IQR = interquartile range, SD = standard deviation, BMI = body mass index, HRT = hormone replacement therapy.

### Clinical characteristics of the “TS group”

Characteristics of patients with TS are presented in Table 2. The median age at diagnosis of TS was 10 years [IQR 4; 15]. Growth hormone treatment had been administered to 71.7% of patients and puberty had been induced in 68.7% of the patients at a mean age of 14.3 years (± 1.9 years).

**Table 2:** Clinical characteristics of the “TS group” (n = 99).

	**“TS group” n = 99**	
**Median age at diagnosis (yr., IQR)**	99	10 [4;15]
**Karyotype (n, %)**	99	
45,X		35 (35.4)
45,X/46,XX mosaicism		16 (16.2)
45,X/46,XY		12 (12.1)
Other mosaicisms		30 (30.3)
X structural anomalies		6 (6.1)
**Past growth hormone treatment (n, %)**	99	71 (71.7)
**Pubertal induction (n, %)**	98	68 (69.4)
Mean age at initiation (yr., IQR)		14.3 ± 1.9
**Heart or aortic anomaly (n, %)**	96	42 (43.8)
Isolated aortic bicuspidy	42	13 (31.0)
Isolated aortic coarctation	42	3 (7.1)
Isolated aortic root dilation	42	11 (26.2)
Aortic valve stenosis	42	1 (2.4)
Combined anomalies	42	14 (33.3)
**History of heart/vascular surgery**	41	11 (26.8)
**Median aortic root index (mm/m** ^**2**^ **)**	96	19.3 [17.5;21.4]
**Hearing impairment (n,%)**	98	36 (36.7)
Use of hearing aids	36	15 (46.9)
**Autoimmune disease (n,%)**	99	29 (29.9)
Hypothyroidism	29	23 (79.3)
Type 1 diabetes	29	0
Coeliac disease	29	5 (17.2)
Vitiligo	29	1 (3.4)

Abbreviations: TS = Turner syndrome, Yrs. = years, IQR = interquartile range.

#### Etiologies of POI

In the “POI group,” the etiologies of POI were respectively X autosomal translocation (n = 1), *FMR1* premutation (n = 2), chemotherapy (n = 6), bilateral ovarian surgery (n = 2), autoimmunity (n = 17), pathogenic gene variants (n = 20) and variants of unknown significance (n = 5). The remaining cases were classified as idiopathic (n = 53; 50%).

#### Relationships and sexual experience

Compared to those from the “POI group,” more patients from the “TS group” were single (41.4% vs 27.6%, *P* = .038), childless (81.6% vs 50.9%, *P* < .0001) or had never had intercourse (21.2% vs 6.6%, *P* = .002) (Table 1). Among those with past sexual activity, the median age at first intercourse was higher in the “TS group” (20 years [IQR 19; 23] vs 19 years [IQR 17; 21], *P* = .011) and they had fewer partners (20% had six or more partners vs 40% in the “POI group,” *P* = .025).

### Results of FSFI-6 questionnaires

#### Analysis in the overall cohort

FSFI-6 scores are presented in Table 3. In the “TS group,” 49.5% of patients had a score below 19 [CI95: 39.3–59.7] compared to 43.4% in the “POI group” [CI95: 33.5–53.4]. This difference is not statistically significant (*P* = .382). Regarding the sub-scores, the only statistically significant difference concerned the frequency of orgasms. In the “POI group,” 50.9% of women reported having had orgasms more than half the time or always during sexual stimulation or intercourse vs 36.4% in the “TS group” (*P* = .008). A subanalysis was performed in order to compare the FSFI total scores according to the patients’ karyotype: homogenous 45, X versus mosaic in the whole Turner’s population. No statistical differences were identified between the two groups of patients (data in Appendix 1).

**Table 3:** FSFI-6 responses in the overall cohort (n = 205).

	**“TS group” n = 99**	**“POI group” n = 106**	***P*-value**
**Overall sexual function alteration (FSFI-6 score < 19)**	49 (49.5)	46 (43.4)	.382^a^
**1. Over the past 4 weeks, how would you rate your level (degree) of sexual desire or interest?**			.247^b^
Very low or none at all	20 (20.2)	12 (11.3)	
Low	21 (21.2)	28 (26.4)	
Moderate	35 (35.4)	43 (40.6)	
High	22 (22.2)	19 (17.9)	
Very high	1 (1.0)	4 (3.8)	
**2. Over the past 4 weeks, how would you rate your level of sexual arousal (“turn on”) during sexual activity or intercourse?**			.631^a^
No sexual activity	19 (19.2)	12 (11.3)	
Very low or none at all	8 (8.1)	11 (10.4)	
Low	9 (9.1)	12 (11.3)	
Moderate	25 (25.3)	24 (22.6)	
High	30 (30.3)	35 (33.0)	
Very high	8 (8.1)	12 (11.3)	
**3. Over the past 4 weeks, how often did you become lubricated (“wet”) during sexual activity or intercourse?**			.427^a^
No sexual activity	24 (24.2)	18 (17.0)	
Almost never or never	7 (7.1)	12 (11.3)	
A few times (less than half the time)	9 (9.1)	6 (5.7)	
Sometimes (about half the time)	11 (11.1)	15 (14.2)	
Most times (more than half the time)	20 (20.2)	17 (16.0)	
Almost always or always	28 (28.3)	38 (35.8)	
**4. Over the past 4 weeks, when you had sexual stimulation or intercourse, how often did you reach orgasm (“climax”)?**			.008^a^
No sexual activity	28 (28.3)	19 (17.9)	
Almost never or never	11 (11.1)	11 (10.4)	
A few times (less than half the time)	9 (9.1)	4 (3.8)	
Sometimes (about half the time)	15 (15.2)	18 (17.0)	
Most times (more than half the time)	22 (22.2)	16 (15.1)	
Almost always or always	14 (14.1)	38 (35.8)	
**5. Over the past 4 weeks, how satisfied have you been with your overall sexual life?**			.093^a^
Very dissatisfied	13 (13.1)	15 (14.2)	
Moderately dissatisfied	6 (6.1)	14 (13.2)	
About equally satisfied and dissatisfied	29 (29.3)	29 (27.4)	
Moderately satisfied	27 (27.3)	15 (14.2)	
Very satisfied	24 (24.2)	33 (31.1)	
**6. Over the past 4 weeks, how often did you experience discomfort or pain during vaginal penetration?**			.411^a^
No sexual activity	32 (32.3)	23 (21.7)	
Almost always or always	5 (5.1)	11 (10.4)	
Most times (more than half the time)	6 (6.1)	8 (7.5)	
Sometimes (about half the time)	11 (11.1)	16 (15.1)	
A few times (less than half the time)	22 (22.2)	21 (19.8)	
Almost never or never	23 (23.2)	27 (25.5)	

Depending on the distribution of the variable, different tests were used:

a Chi-square test.

b Fisher’s test.

Abbreviations: TS = Turner syndrome, POI=Premature ovarian insufficiency.

#### Analysis focussing on sexually active patients

A secondary analysis focussed on sexually active patients. Indeed, in four out of the six questions in the FSFI-6 questionnaire, the answer may be “no sexual activity”. Participants were defined as sexually inactive during the past 4 weeks and therefore excluded from this secondary analysis if they had answered “no sexual activity” in at least two out of the four questions. Thirty patients reported “no sexual activity” in all four questions and 26 reported “no sexual activity” at least in two out of the four questions. This secondary analysis included 159 patients: 72 (72.7%) in the “TS group” and 87 (82.1%) in the “POI group.” Among sexually active women, an FSFI-6 score below 19 was observed in 30.6% [CI95: 20.2–42.5] of the “TS group” and in 31% [CI95: 21.6–41.9] of the “POI group.” This difference is not statistically different. Among the patients with TS, no statistical differences were identified according to the patients’ karyotype (data in Appendix 1).

#### Univariate and multivariate analyses according to patients’ characteristics

The association between patients’ characteristics and an FSFI-6 score below 19 was investigated using univariate and multivariate logistic regression analyses in the entire cohort and in both groups, “TS” and “POI.” The “age” variable was forced into each multivariate model. Missing data on the selected variables underwent multiple imputation for the multivariate analysis. In the overall cohort, as well as in both groups, no association was found between impaired sexual function and age nor the use of HRT. None of the comorbidities of TS was associated with an increased risk of impaired sexual function.

However, a significant association between relationship status and sexual function impairment was identified in each analysis. Indeed, single patients had a higher risk of experiencing impaired sexual function compared to patients in a relationship. This risk was even higher in the “TS group” (OR = 8.82 [CI95: 2.91-26.70], *P* = .0001) than in the “POI group” (OR = 3.50 [CI95: 1.40-8.73], *P* = .007).

## Discussion

The main goal of SEXATURN study was to compare sexual function of patients with TS to that of patients with POI from other etiologies. Our results show lower quantitive data in women with TS but similar qualitative data between the two populations.

Several studies comparing TS patients to the general population have found a decrease in sexual activity among patients with TS.^18–22^ Cardona Attard et al. showed that British women with TS have a delayed mean age at first vaginal sexual intercourse compared to women with non-TS POI.^23^ SEXATURN shows a significant decrease in all quantitative data of sexual activity among patients with TS, compared to patients with non-TS POI: less frequent intercourse, later onset of sexual activity, fewer partners. Several features of TS, such as hearing impairments or the tendency toward shyness and social anxiety may hinder TS patients’ ability to connect with sexual partners. Furthermore, physical traits like short stature, overweight or obesity could impact the patients’ self-esteem.^5^^,^^24^

SEXATURN study provides not only quantitative but also qualitative assessment of sexual function. We observed a high prevalence of impairment, reaching 49.5 % in the overall cohort of TS and 30.6 % of the sexually active TS patients. In a recent study by Tarantino et al. involving 29 patients with TS with a mean age of 31.4 ± 8.9 years, 51.7% were sexually active.^17^ Using the FSFI questionnaire, 57% of FSD were reported. In SEXATURN study including a larger number of patients, 72.7% were sexually active and the percentage of impairment of sexual function was lower.

In our study, the prevalence of impaired sexual function was slightly higher in women with TS compared to those with POI from other causes. However, the difference was not statistically significant. Therefore, our data do not confirm our initial hypothesis of a higher rate of impaired sexual function in patients with TS compared to the ones with POI. The multivariate analysis in SEXATURN showed that the relationship status is a major protective factor against impaired sexual function in both groups, but even more in the “TS group” than in the “POI group” (OR = 8.82 [CI95: 2.91-26.70] vs 3.50 [CI95: 1.40-8.73]). Several studies have shown that although women with TS are less likely to be in relationships than the general population, those in long-term relationships report high levels of satisfaction with their intimate and sexual lives, whereas single patients show significantly impaired sexual function, particularly regarding desire.^19^^,^^20^^,^^28^ In SEXATURN, 59% of the “TS group” were in a relationship, compared to a usual percentage ranging from 29% to 39% in other cohorts of TS patients.^5^^,^^19^^,^^20^^,^^23^^,^^28^ This percentage may have increased FSFI-6 scores in the “TS group.” Another hypothesis for the lack of statistically significant difference could be related to the psychological profiles of patients with TS, who tend to underreport their difficulties. Some studies have shown that they may provide socially desirable responses.^29^^,^^30^ On the other hand, POI by itself has a significant impact on overall psychological quality of life, with a higher occurrence of anxiety, depression, somatization, and diminished self-esteem.^31^ Several studies have even suggested that in women with POI, psychological factors may have a larger impact than physical factors, in the occurrence of sexual dysfunctions.^32–34^

In order to interpret our results, we searched for data assessing the prevalence of Female Sexual Dysfunction (FSD) in the general population^30–34^ or in patients with chronic diseases.^35–37^ In the general population, FSD in previously published studies ranges from 30% to 50% in women of all ages.^35–39^ Our results fall within this cited range. However, percentages in the general population may be biased, as sexually inactive or single women are excluded, in some studies. Furthermore, data concerning anxiety, depression or the presence of chronic diseases are lacking. Several studies have reported an increased prevalence of FSD in women with chronic pathologies.^40–42^ Studies from the general population or women with chronic pathologies are heterogeneous and therefore limit the extent to which comparisons with our findings are accurate.

A strength of SEXATURN study is based on the populations studied. Our patients are well phenotyped in a single reference center of rare diseases. Cardiovascular, autoimmune, and ENT comorbidites were collected for all patients with TS. The median age of the overall cohort at the time of the study was 37 years old, providing a good assessment of their past and present sexual experiences. Furthermore, the use of HRT was similar in both groups (89.4% in the overall cohort).

We used the FSFI-6 questionnaire which is derived from the FSFI, the gold standard in research evaluating sexual function.^25^^,^^26^ In most previous studies including TS patients, response rates were below 50%.^21–23^^,^^28^ In SEXATURN, the response rates were 67% of patients in the “TS group” and 77% in the “POI group,” respectively. These higher response rates may be related to the fact that we used the short version of the FSFI which is easier to fill. A second explanation relies on our two-step methodology including a preliminary phone call to address the patient’s questions before submitting the questionnaires. Another strength is that we performed a subanalysis in sexually active women. Our study shows a non-significant difference between the POI and the non-POI group.

However, SEXATURN study also has some weaknesses. First, reaching the patients over the phone was difficult. This may have selected women more at ease discussing their sexuality and/or with less severe conditions, particularly among patients with TS. In our study, we observed a high percentage of sexually active women in the group with TS compared to other studies.^16^^,^^17^^,^^19^^,^^22^ This could be explained by the fact that some women with TS refusing to answer the questionnaire were not sexually active and/or were not motivated by a study exploring sexual activity. These issues have been raised by some patients during the first phone-call they received when the study was proposed. Furthermore, the percentage of patients with mosaicism in our population is 65%. This percentage is slightly higher than the percentages observed in different studies including patients with TS.^1^^,^^17^ As patients with mosaicism have a milder phenotype than patients with 45,X homogenous karyotype,^43^ the percentage of women with mosaicism could explain higher FSFI-6 scores in the TS group than expected. However, in our study, no difference was observed for the FSFI scores between patients with 45,X homogenous karyotypes or patients with 46,XX/45,X mosaicisms. Very few studies have evaluated so far the sexual function of patients with TS, according to their respective karyotypes. Finally, the FSFI-6 questionnaire has some limitations.^44^ While it is easy to fill, it cannot be used for diagnostic purposes of FSD,^45^^,^^46^ as it does not include a distress scale. Furthermore its evaluation is limited to the 4 weeks prior to the filling of the questionnaire. The FSFI-6 does not evaluate sexual experience, fantasies, expectations, or interpersonal functioning. The heteronormative formulation of the questions is not appropriate to assess sexual function of women who are single, in same-sex relationships, or having non-penetrative sex. Another limit in our study using this tool is that while sexual inactivity in women with TS is an important outcome, the FSFI-6 data concerning sexually inactive women cannot be assessed. Furthermore, in our study the number of participants reporting “no sexual activity” varied substantially across items, but the FSFI-6 does not provide data concerning the different behavioral situations. Finally, in our study, to improve the response rate, data concerning sexual function were anonymously collected. For instance, if Tarantino et al. were able to obtain data on autoeroticism, the design of our study prevented from any follow-up inquiries.^17^ Sexual inactivity in patients with TS is an important subject and studies addressing this issue in large cohorts of women with TS should be performed.

## Conclusion

In SEXATURN study, a large proportion of impaired sexual function was found among patients with POI, whether or not related to TS. The FSFI-6 questionnaire, although it is not a diagnostic tool of FSD, could be used routinely for screening patients in order to refer them to sexology consultations. No predictive factors were identified among the comorbidities in TS patients associated with impaired sexual function. Further studies are needed to explore the reasons for impaired sexual function in these patients and evaluate the benefits of taking care of sexual function on quality of life. Our data illustrate the urgent need for sexology and psychological consultations within the multidisciplinary care of patients with TS or POI from other causes.

## Supplementary Material

7_SEXATURN_appendix1_ok_qfag074

## Subjects and methods

“SEXATURN” (SEXuAlity in patients with TURNer syndrome) is a monocentric, cross-sectional study including patients from the “CRESCENDO” center for rare endocrine diseases of growth and development, located in the Endocrine unit at Saint-Antoine Hospital, Assistance Publique – Hôpitaux de Paris (AP-HP), Sorbonne-University. This center is affiliated to the “FIRENDO” national network for Rare Endocrine Diseases and to the Endo-ERN network (endo-ern.eu id 739527).

The study protocol was approved a randomly assigned ethics committee (Comité de Protection des Personnes Est-II) on September 27, 2020 (IDRCB 2020-A01768-31). In compliance with French law, the study complies with the reference methodology MR-003 published by the National Commission for Computing and Liberties (CNIL) and with the declaration of Helsinki. This study is registered on the website ClinicalTrial.gov (NCT05223621).

## Statistical analysis

Data management and statistical analysis were carried out by the Clinical Research Unit of East-Paris (URC-Est) Sorbonne University, from the Delegation for Clinical Research and Innovation (DRCI), Assistance Publique-Hôpitaux de Paris.

## Attestation statement

The subjects in this trial have not concomitantly been involved in other randomized.

Trials. Data regarding any of the subjects in the study has not been previously published. Data will be made available to the editors of the journal for review or query upon request.

## Data sharing statement

Study data cannot be shared due to regulatory restrictions that apply to the availability of data generated and analyzed during this study to preserve patient confidentiality and according to the General Data Protection Regulation.
