# Supplementary material for: SEXATURN: sexual function of patients with Turner syndrome compared to that of women with premature ovarian insufficiency
Source: Sex Med. 2026 Aug 17;14(5):qfag074. doi: 10.1093/sexmed/qfag074 (PMC13480476; doi:10.1093/sexmed/qfag074)
Supplement: Supplementary file 1 [file 7_sexaturn_appendix1_ok_qfag074.docx]

Appendix 1: comparison of FSFI-6 scores among patients with TS, according to their karyotypes

| Outcome | TS n=99 | | group 45,X n=35 | | mosaic n=64 | | p-value |
| --- | --- | --- | --- | --- | --- | --- | --- |
| **FSFI-6 score among TS group (median, IQR)** | 99 | 19.0 (9.0 ; 23.0) | 35 | 17.0 (5.0 ; 23.0) | 64 | 20.0 (12.0 ; 23.0) | 0.231^$^ |
| **Overall impairment of sexual function (FSFI-6 score < 19) among TS group (n, %)** | 99 |  | 35 |  | 64 |  | 0.26^£^ |
| No |  | 50 (50.5) |  | 15 (42.9) |  | 35 (54.7) |  |
| Yes |  | 49 (49.5) |  | 20 (57.1) |  | 29 (45.3) |  |
| **Sexual activity (n, %)** | 99 |  | 35 |  | 64 |  |  |
| No |  | 27 (27.3) |  | 12 (34.3) |  | 15 (23.4) |  |
| Yes |  | 72 (72.7) |  | 23 (65.7) |  | 49 (76.6) |  |
| **FSFI-6 score among TS group with sexual activity  (median, IQR)** | 72 | 21.5 (18.0 ; 24.0) | 23 | 21.0 (17.0 ; 24.0) | 49 | 22.0 (18.0 ; 24.0) | 0.89^$^ |
| **Overall impairment of sexual function (FSFI-6 score < 19) among TS group with sexual activity (n, %)** | 72 |  | 23 |  | 49 |  | 0.594^£^ |
| No |  | 50 (69.4) |  | 15 (65.2) |  | 35 (71.4) |  |
| Yes |  | 22 (30.6) |  | 8 (34.8) |  | 14 (28.6) |  |

TS = Turner syndrome. IQR = interquartile range. Depending on the distribution of the variable, different tests were used: ^£^ chi-square test, ^$^ Wilcoxon-Mann-Whitney test.
